# Dataset on the nurses’ knowledge, attitude and practice towards palliative care

**DOI:** 10.1016/j.dib.2018.11.133

**Published:** 2018-12-05

**Authors:** Amir Hosein Farmani, Seyed Reza Mirhafez, Ali Kavosi, Azam Moghadam Pasha, Ahmad Jamali nasab, Gholamreza Mohammadi, Vahid Moeini, Mohammad Reza Aryayi Far, Ali Movahedi

**Affiliations:** a Students Research Committee, Neyshabur University of Medical Sciences, Neyshabur, Iran; b Department of Basic Medical Sciences, Neyshabur University of Medical Sciences, Neyshabur, Iran; c Nursing Research Center, Golestan University of Medical Sciences, Gorgan, Iran; d 22 Bahman Hospital, Neyshabur University of Medical Sciences, Neyshabur, Iran; e Department of Anesthesia and Operating Room Nursing, Neyshabur University of Medical Sciences, Neyshabur, Iran

**Keywords:** Attitude, Knowledge, Practice, Nurses, Palliative care

## Abstract

When a patient enters the end stage of life threatening disease like cancer, treatment of pain and other symptoms must be considered to preserve quality of life (Gielen et al., 2011) [1]. Nurses have an important role in the care of patients who suffered from life threatening diseases. End of life cares is one of the routine activities of nurses (Gott et al., 2012) [2]. We surveyed knowledge, attitude and practice of nurses who worked in the hospitals of Neyshabur University of Medical Sciences towards palliative care from January 2016 to May 2016. A self-administered Persian questionnaire was used for data collection. The attitude scale was adopted from Frommelt Attitude toward Care of the Dying (Frommelt, 1991) and the knowledge questions were adopted from the Palliative Care Quiz for Nursing (Ross et al., 1996). The practice questions were also adopted from different related studies. Data analysis was performed by SPSS Statistics software for windows version 16. Our study showed that majority of nurses had favorable attitude but poor knowledge and practice towards palliative care. The results emphasize the importance and need for developing palliative care services in our hospitals.

**Specifications table**

**Table:** 

Subject area	Nursing and Health Professions, clinical research
More specific subject area	Knowledge, attitude and practice of nurses towards palliative care
Type of data	Table, text file
How data were acquired	Survey, paper-based
Data format	Categorized, analyzed
Experimental factors	Data collected in a cross sectional survey research design study
Experimental features	Survey collected data from nurses in Neyshabur University of Medical Sciences. We report the survey data for nurse׳s knowledges, attitudes, and practices towards palliative care.
Data source location	Neyshabur, Iran
Data accessibility	Data within this article

**Value of the data**

• Data and survey instrument questions can be compared with or inform other studies.
• Data emphasize the need for developing palliative care services in Iran.
• Nurses have a prominent role in end of life care. Palliative care needs to become a part of nursing and medical school curricula as well as continuing nursing and medical education program offerings.

## 1 Data

Socio-demographic characteristics of nurses were summarized in Table 1. The total number of nurses was 117 and the response rate was 116. Mean age of the nurses was 32.37 ± 8.47 years (range from 22 to 50). The mean±SD (standard deviation) of knowledge score of nurses in this study were 7.15 ± 2.91 (range 3–13). As shown in the Table 2, from the total respondents, 22 nurses (19.3%), 64 nurses (56.1%) and 28 nurses (24.6%) had good knowledge, moderate knowledge and poor knowledge towards palliative care, respectively. Attitude of nurses towards palliative care was measured by Frommelt Attitude toward Care of the Dying scale. The total attitude score of nurses in this study was 74.98 ± 8.18 (range 53–110). From the total respondents, 99 nurses (85.3%) and 17 nurses (14.7%) had favorable attitude and unfavorable attitude toward palliative care, respectively (see Table 3). The total practice score of nurses in this study was 17.22 ± 8.19 (range 3–34). Also the results showed only 7 (6.3%) of the respondents had good practice towards palliative care (see Table 4). Association between socio-demographic variables such as age, gender, level of education and work experience and nurse׳s knowledge, attitude and practices towards palliative care was analyzed by Chi square test, Fisher exact test and univariate & multivariate analysis. We found that ward and level of education had a significant association with practice of nurses toward palliative care. The nurses who working in intensive care units had better practice toward palliative care. The nurses who had master degree had better practice toward palliative care. Level of education also had a significant association with attitude of nurses toward palliative care (see Table 5). The result of this study suggested that majority of nurses had favorable attitude but poor knowledge and practice towards palliative care. These results emphasize the importance and need for developing palliative care services in our hospitals. Nurses have a prominent role in patient׳s end of life care. Palliative care needs to become a part of nursing and medical school curricula as well as continuing nursing and medical education program offerings.

**Table 1:** Socio-demographic characteristics of nurses at selected hospitals in Neyshabur, February 2016.

Characteristics	Frequency	Percentage
Age		
20–30 years	59	50.7
31–40 years	33	28.4
41–50 years	24	20.7
Sex		
Female	92	79.3
Male	24	20.7
Institution		
Hakim	63	54.3
22 Bahman	53	45.7
Level of education		
Diploma	1	0.9
Associate degree	3	2.6
Bachelor׳s degree	106	93.0
Master degree	4	3.5
Working experience		
Less than 5 years	56	48.3
5–10 years	19	16.4
11–15 years	16	13.8
16–20 years	8	6.9
>20 years	17	14.7
Ward		
Medical	8	7.1
Surgical	21	18.8
Emergency	11	9.8
Cardiac	7	6.2
ICU	30	26.8
Neonates	4	3.6
Polyclinic	4	3.6
Nursing office	4	3.6
Maternity	14	12.5
Chemotherapy	5	4.5
Dialysis unit	4	3.6
Palliative care training		
Yes	27	23.3
No	89	76.7

ICU: intensive care unit

**Table 2:** Distribution of nurse׳s knowledge towards palliative care at selected hospitals in Neyshabur, February 2016.

No.	Question	Yes N (%)	No N (%)	Don’t know N (%)
1	Do you know the definition of palliative care?	51 (44.0)	65 (56.0)	–
2	Palliative care is only appropriate in situations of a downhill trajectory or deterioration in conditions.	12 (10.3)	90 (77.7)	14 (12.0)
3	The extent of the disease determines the method of pain treatment.	73 (62.9)	30 (25.9)	13 (11.2)
4	Adjuvant therapies are important in the pain management.	82 (70.7)	21 (18.1)	13 (11.2)
5	Drug addiction is a major problem when morphine is used in long-term for the pain management.	64 (55.7)	31 (26.2)	21 (18.1)
6	The provisions of palliative care require emotional detachment.	76 (66.1)	20 (16.9)	20 (16.9)
7	During the terminal stages of an illness, drugs that can cause respiratory depression are appropriate for the treatment of severe dyspnea.	28 (24.1)	36 (31.1)	52 (44.8)
8	The philosophy of palliative care is compatible with aggressive treatment.	37 (32.5)	51 (43.4)	28 (24.1)
9	The use of placebos is appropriate in the treatment of some types of pain.	76 (66.1)	20 (16.9)	20(16.9)
10	Meperidine is not an effective analgesic for the control of chronic pain.	54 (47.4)	50 (42.3)	12 (10.3)
11	The accumulation of losses renders burnout Inevitable for those who work in palliative care.	49 (41.5)	47 (41.6)	20 (16.9)
12	Manifestations of chronic pain are different from those of acute pain.	78 (68.0)	18 (15.1)	20 (16.9)
13	Terminally ill patients have the right to choose “Do not resuscitate” (DNR).	60 (52.0)	36 (31.1)	20 (16.9)
14	Terminally ill patients should be encouraged to have hope against all odds.	83 (71.5)	20 (16.9)	13 (11.6)

**Table 3:** Distribution of nurses attitude towards palliative care at selected hospitals in Neyshabur, February 2016.

No.	Question	SD (%)	D (%)	U (%)	A (%)	SA (%)
1	Palliative care is given only for dying patient.	29 (26.1)	39 (35.1)	26 (23.4)	16 (14.4)	1 (0.9)
2	As a patient nears death; the nurse should withdraw from his/her involvement with the patient.	34 (30.6)	33 (29.7)	22 (19.8)	15 (13.5)	7 (6.3)
3	Giving nursing care to the chronically sick patient is a worthwhile learning experience.	5 (4.5)	5 (4.5)	20 (18.0)	64 (57.7)	17 (15.3)
4	It is beneficial for the chronically sick person to verbalize his/her feelings.	4 (3.6)	4 (3.6)	15 (13.6)	70 (63.6)	17 (15.5)
5	Family members who stay close to a dying person often interfere with a professional׳s job with the patient.	4 (3.6)	25 (22.5)	12 (10.8)	50 (45.0)	20 (18.0)
6	The length of time required to give nursing care to a dying person would frustrate me.	20 (18.0)	45 (40.5)	23 (20.7)	14 (12.6)	9 (8.1)
7	Families should be concerned about helping their dying member make the best of his/her remaining life.	10 (9.1)	6 (5.5)	21 (19.1)	51 (46.4)	22 (20.0)
8	Family should maintain as normal an environment as possible for their dying member.	5 (4.5)	6 (5.5)	14 (12.7)	53 (48.2)	32 (29.1)
9	The nurse should not be the one to talk about death with the dying person.	4 (3.7)	18 (16.5)	23 (21.1)	46 (42.2)	18 (16.5)
10	The family should be involved in the physical care of the dying person.	7 (6.6)	4 (3.8)	13 (12.3)	59 (55.7)	23 (21.7)
11	It is difficult to form a close relationship with the family of a dying member.	6 (5.6)	29 (26.9)	21 (19.4)	36 (33.3)	16 (14.8)
12	There are times when death is welcomed by the dying person.	6 (5.6)	21 (19.4)	34 (31.5)	35 (32.4)	12 (11.1)
13	Nursing care for the patient׳s family should continue throughout the period of grief and bereavement.	9 (8.3)	21 (19.4)	30 (27.4)	39 (35.8)	10 (9.2)
14	The dying person and his/her family should be the in-charge decision makers.	5 (4.6)	25 (22.9)	33 (30.3)	36 (33.0)	10 (9.2)
15	Addiction to pain relieving medication should not be a nursing concern when dealing with a dying person.	4 (3.7)	16 (14.7)	22 (20.2)	43 (39.4)	24 (22.0)
16	Nursing care should extend to the family of the dying person.	6 (5.7)	17 (16.0)	20 (18.9)	46 (43.4)	17 (16.0)
17	When a patient asks, “Nurse am I dying?׳ I think it is best to change the Subject to something cheerful.	6 (5.5)	23 (20.9)	25 (22.7)	43 (39.1)	13 (11.8)
18	I am afraid to become friends with chronically sick and dying patients.	18 (16.4)	45 (40.9)	23 (20.9)	20 (18.2)	4 (3.6)
19	I would be uncomfortable if I entered the room of a terminally ill person and found him/her crying.	4 (3.6)	16 (14.5)	18 (16.4)	55 (50.0)	17 (15.5)
20	I would be uncomfortable talking about impending death with the dying Person.	3 (2.8)	14 (12.8)	16 (14.7)	61 (56.0)	15 (13.8)
21	It is possible for nurses to help patients prepare for death.	11 (10.1)	21 (19.3)	36 (33.0)	32 (29.4)	9 (8.3)
22	Death is not the worst thing that can happen to a person.	5 (4.6)	20 (18.3)	26 (23.9)	37 (33.9)	21 (19.3)
23	I would feel like running away when the person actually died.	15 (13.6)	36 (32.7)	28 (25.5)	24 (21.8)	7 (6.4)
24	I would not want to be assigned to care for a dying person.	12 (10.9)	33 (30.0)	25 (22.7)	27 (24.5)	13 (11.8)

SD: strongly disagree, D: disagree, U: uncertain, A: agree, SA: strongly agree.

**Table 4:** Practice of nurses towards palliative care at selected hospitals in Neyshabur, February 2016.

No.	Question	Multiple response	Yes N (%)	No N (%)
1	Initiate palliative care discussion:	During diagnosis	85 (75.9)	27 (24.1)
		When the disease progress	37 (33.0)	75 (67.0)
		At the end of life	16 (14.3)	96 (85.7)
2	Do you inform terminally ill patient about their diagnosis?	Yes	61 (54.5)	51 (45.5)
		Depending on family׳s wish	46 (41.1)	66 (58.9)
3	Factors considered when dealing with terminally ill patient:	Spiritual	80 (72.1)	31 (27.9)
		Medical situation	64 (57.7)	47 (42.3)
		Cultural	43 (38.7)	68 (61.3)
		Psychological	49 (44.1)	62 (55.9)
4	Address spiritual issue:	Connect with spiritual counselor	33 (29.7)	78 (70.3)
		Listen with empathy	73 (65.8)	38 (34.2)
		Impose your own view	4 (3.6)	107 (96.4)
		Understand patient reaction	50 (45.0)	61 (55.0)
5	Cultural assessment during patient care should include:	Truth telling and decision making	55 (49.5)	56 (50.5)
		Preference regarding disclosure of information	5 (4.5)	106 (95.5)
		Dietary preference	26 (23.4)	85 (76.6)
		Language, family communication	52 (46.8)	59 (53.2)
		Perspective on death, suffering & grieving	38 (34.2)	73 (65.8)
6	Addressing psychological:	Emotional support	83 (74.8)	28 (25.2)
		Counseling the patient	53 (47.7)	58 (52.3)
		Hiding the truth	5 (4.5)	106 (95.5)
7	Whom do you involve in decision making?	Patient	76 (68.5)	35 (31.5)
		Family	67 (60.4)	44 (39.6)
		My own	45 (40.5)	66 (59.5)
		Other health professional	42 (37.8)	69 (62.2)
8	How do you perceived terminally ill patient concern or question?	Patient right	64 (57.7)	47 (42.3)
		Treat	55 (49.5)	56 (50.5)
		Doubting your professionalism	22 (19.8)	89 (80.2)
		Attention seeking behavior	50 (45.0)	61 (55.0)
9	Communication to the family of terminally ill patient depends on:	Family׳s ability to assimilate	63 (56.8)	48 (43.2)
		Their involvement in decision making	68 (61.3)	43 (38.7)
		Your willingness to disclose information	30 (27.0)	81 (73.0)
10	Commonly use medication in your practice for severe pain?	Paracetamol/Ibuprofen	15 (13.5)	96 (86.5)
		Codeine	17 (15.3)	94 (84.7)
		Morphine	87 (78.4)	24 (21.6)
11	How do you assess patient pain?	Grade with face	52 (46.8)	59 (53.2)
		Intensity	73 (65.8)	38 (34.2)
		Location	62 (55.9)	49 (44.1)
		Quality	68 (61.3)	43 (38.7)

**Table 5:** The association of socio-demographic characteristics and attitude, knowledge and practice of nurses toward palliative care at selected hospitals in Neyshabur, February 2016.

Variables	Attitude (*P* value)	Knowledge (*P* value)	Practice (*P* value)
Ward	*P* > 0.57	*P* > 0.12	*P* < 0.001
Level of education	*P* >0.005	*P* > 0.47	*P* < 0.03

## 2 Experimental design, materials and methods

A cross-sectional study was conducted with nurses at 2 Hospitals related to Neyshabur University of Medical Sciences in Neyshabur city, northeastern Iran, from January 2016 to May 2016. The nurses who working in outpatient departments and clinical wards of the hospitals were recruited to participate in the study with at least 1 year job experience. However, nurses working in the central sterilization supply department, operating room and delivery rooms were excluded. The sample size according to the similar study [5] was determined 116 nurses. Samples were selected as classified random sampling. A self-administered Persian questionnaire was used for data collection. The attitude scale was adopted from Frommelt Attitude toward Care of the Dying scale [3] which was consist of 24 items. The tool has a 5 point Likert scale. This was used to represent people׳s attitudes to a topic scored on 5 point scale, i.e. 1 (Strongly Disagree), 2 (Disagree), 3 (Uncertain), 4 (Agree) 5 (Strongly Agree). Twelve of the questions were worded positively and twelve were worded negatively. The score of negative questions was reversed. The attitude was favorable if participants gained the score at least 50% of the total attitude score. The knowledge questions were adopted from the palliative care quiz for nursing using questions with Yes (Score 1), No, and Don’t know (Score 0) answers [4]. The knowledge was good if participants get the score at least 75% of total knowledge score. The knowledge score between 75% and 25% was defined as moderate knowledge and less than 25% as poor knowledge. The practice questions were also adopted from different related studies which was includes 11 practical questions. The practice was good if participants gained the score more than 75% of total practice score. Data collection was done by two Educational Supervisor nurses in the two hospitals. Data analysis was performed by SPSS Statistics software for Windows version 16 (IBMCorp., Armonk, NY). The study was approved by Ethics Committee of the Neyshabur University of Medical sciences (ethical approval number: IR.NUMS.REC.1394.11). Verbal consent was obtained from each participants, and participant׳s anonymity and confidentiality was kept. The respondents had the right to withdraw from the study, at any stage.
